# Intake of n-3 LCPUFA and trans-fatty acids is unrelated to development in body mass index and body fat among children

**DOI:** 10.1186/s40795-021-00493-5

**Published:** 2022-01-03

**Authors:** Xuan Ren, Sofus Christian Larsen, Lotte Lauritzen, Jeanett Friis Rohde, Lars Bo Andersen, Anna Bugge, Britt Wang Jensen, Ina Olmer Specht, Berit Lilienthal Heitmann

**Affiliations:** 1 Research Unit for Dietary Studies at The Parker Institute, Bispebjerg and Frederiksberg Hospital, Frederiksberg, Denmark; 2 Department of Nutrition, Exercise and Sports, Paediatric and International Nutrition, University of Copenhagen, Copenhagen, Denmark; 3 Department of Education, Arts and Sport, Western Norway University of Applied Sciences, Sogndal, Norway; 4 Department of Midwifery, Physiotherapy, Occupational Therapy and Psychomotor Therapy, University College Copenhagen, Copenhagen, Denmark; 5 Center for Clinical Research and Prevention, Bispebjerg and Frederiksberg Hospital, Frederiksberg, Denmark; 6 The Boden Group, Faculty of Medicine and Health, Sydney University, Sydney, Australia; 7 Department of Public Health, Section for General Medicine, University of Copenhagen, Copenhagen, Denmark

**Keywords:** fatty acids, children, BMI, body fat, substitution

## Abstract

**Background:**

The number of children and adolescents with obesity has increased worldwide. Some studies have found an increase in the intake of n-3 long-chain polyunsaturated fatty acid (LCPUFA) to be beneficial for weight and obesity status. The objectives of this study were to examine if intake of trans-fatty acids (TFA) and n-3 LCPUFA at school start was associated with weight and body fat development in the following 3 and 7 years, and if substituting other fats for n-3 LCPUFA in regression models influenced weight and body fat development.

**Methods:**

A total of 285 children (boys:130, girls:155) were included in this study. Weight, height and skinfold thickness (SF) of children were measured at age 6, 9 and 13 years by trained research personnel. Multivariate linear regression models were used to investigate the associations between n-3 LCPUFA or TFA intake and subsequent changes in body mass index (BMI) or SF. To investigate substitution effects, we constructed regression models including information on n-3 LCPUFA and all other energy given components of the diet, except for the nutrient to be substituted (all other fats and specific subgroups; saturated fatty acids (SFAs), monounsaturated fatty acids (MUFAs) and other polyunsaturated fatty acids (PUFAs)).

**Results:**

No significant associations were observed between intake of TFA or n-3 LCPUFA and changes in BMI and SF. Also, results from regression analysis showed substituting other fats for n-3 LCPUFA did not associate with BMI or SF development.

**Conclusion:**

The lack of associations between n-3 LCPUFA and TFA and adiposity suggests that fat composition in the diet does not play a major role in obesity development among school-aged children.

**Supplementary Information:**

The online version contains supplementary material available at 10.1186/s40795-021-00493-5.

## Introduction

In the past decades, the number of children and adolescents with obesity has increased worldwide [1]. Obesity was previously considered a problem only in developed countries, but now it is also on the rise in developing countries [2]. Globally, over 340 million children (around 18% of children) and adolescents between 5-19 are overweight or obese [2]. Several studies suggest that children with obesity in early childhood are more prone to overweight or obesity as teenagers and adults [3, 4]. Furthermore, obesity in childhood is a significant risk factor for several lifestyle diseases [5] and premature death [6]. Therefore, it is essential to identify strategies to prevent childhood obesity [7].

Fat is an important nutrient during childhood, and several studies have examined the effect of different types of fatty acids intake on weight development, but the results have generally been inconsistent [8–13]. Cross-sectional studies have found an association between overweight or obesity and the intake of trans fatty acid (TFA) [12], however a previous study among 5-12 years old children found no association between serum TFAs used as biomarkers of TFA intake, and subsequent 30 months weight gain [13]. Moreover, studies in children have found that a high intake of n-3 long chain polyunsaturated fatty acid (n-3 LCPUFA) or a higher ratio of PUFAs to SFA was related to less visceral obesity and more lean mass as well as a reduced risk of obesity [8, 9, 14]. In contrast, one systematic review demonstrated no association between n-3 LCPUFA and weight development [15]. This inconsistent result may result from a change in fatty acid composition when n-3 LCPUFA intake increases. In this regard a few studies among adults have found beneficial effects of replacing saturated fatty acid (SFA) with monounsaturated fatty acid (MUFA) or polyunsaturated fatty acid (PUFA) on development in body weight and obesity [16, 17].

There is a growing focus on modifying foods in order to replace unhealthy nutrients, e.g. replacing SFA with PUFA, as a strategy to reduce the risk of lifestyle diseases [18]. A previous study has demonstrated that replacing SFA and TFA with PUFA by different kinds of biscuits (low-fat biscuits, butter shortbread biscuits and canola shortbread biscuits) contributed to a significant weight loss and improved serum lipid in adults [19]. However, there is a general lack of studies, which examine the long-term effects of substituting the intake of specific types of fats for subsequent changes in adiposity, especially studies that investigate whether fatty acid replacement may be beneficial in the prevention of weight gain and adiposity development during childhood. Therefore, the present study aimed to investigate associations between the intake of TFA and n-3 LCPUFA among healthy 6-year old children and changes in body mass index (BMI) and skinfold thickness (SF) over the subsequent 3 and 7 years. We further examined associations with statistical substitutions of dietary intake from SFA, MUFA, and other PUFAs for n-3 LCPUFA and if the results are affected by changes in height and puberty stage.

## Subjects and methods

### Subjects

**Fig. 1:**
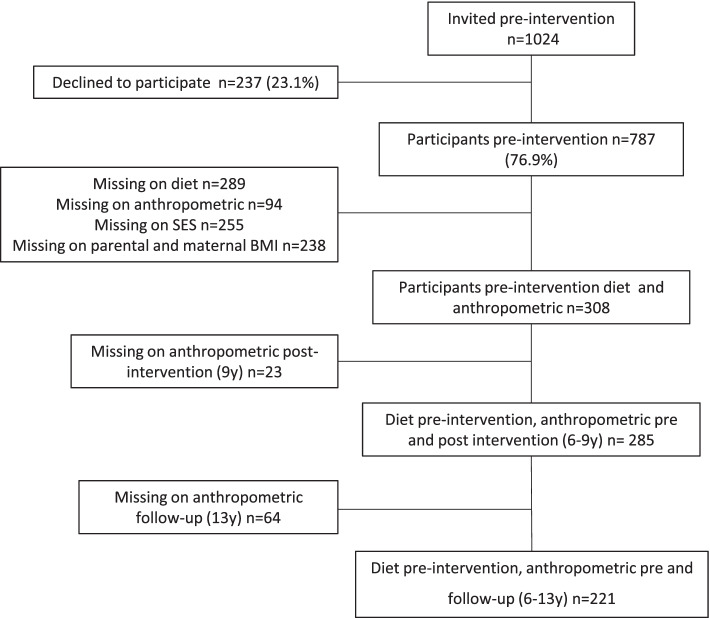
Flow chart of the study population

The data were derived from the Copenhagen School Child Intervention Study (CoSCIS) [20, 21]. In brief, the CoSCIS intervention delivered two additional physical activity exercise lessons per week for the first 3 years of school; improvement of schoolyard environment; selling healthy diet and snacks in school canteens; involvement of parents and health education. Children from 10 schools (27 classes) in the Ballerup municipality where enrolled in the intervention and children from 8 schools (19 classes) in the Tårnby municipality were chosen as controls [22]. In total, 1024 children were invited to participate and 68.46% (intervention: n=411, comparison: n=290) of the children and their parents or caregivers gave written informed consent. Of these, 285 (41%) and 221 (32%) children from both intervention and comparison groups with information on diet at age 6 years (baseline) were followed up at age 9 and 13 years, respectively, for development in BMI and skinfold thickness (Fig. 1). No main effects of the intervention were observed on physical activity, BMI or intake of macronutrients among the children after the 3-year intervention period [21, 23]. Thus, in this study we investigated the children as a cohort, taking the intervention status into account in the analyses.

### Dietary assessment

Information on the children’s dietary intake at both school entry (age 6 years) and grade 3 (age 9 years), was recorded for 7 consecutive days by parents or caregivers. Pre-printed food records were used to collect information on dietary habits [23], and were delivered via the teachers in closed envelopes, which the child brought home [24]. The National Food Institute, Technical University of Denmark developed and slightly modified (e.g. remove all alcoholic beverage) the food records to fit children [25]. According to the typical Danish food pattern the food records included four parts: breakfast; lunch; dinner; and in between snacks. Each meal was divided into subsections with beverage, bread, cereal, vegetables etc. and pre-printed with common foods and drinks and supplemented with an open-ended category [26]. The amount of foods consumed was assessed via household measurement scales and with photo series [24], illustrating portion sizes of common Danish foods in four or six different quantities [24] .

The completed dietary records were scanned by Eyes & Hands version 5.2. Consumptions of nutrients were calculated using the General Intake Estimation System (version 1.000, released 26 February 2010, developed by the National Food Institute, Technical University of Denmark) and the Danish Food Consumption Databank (version 6) [24]. The dietary information included in the present study were total energy intake (MJ), total fat (energy percentage (E%), g/d), SFA (E%, g/d), MUFA (E%, g/d), non-n-3 LCPUFA (E%, g/d), n-3 LCPUFA (E%, g/d), TFA (E%, g/d), protein (E%, g/d) and carbohydrate (E%, g/d) from children aged 6 years only.

### Anthropometry and other measurements

Weight, height and skinfold thickness of children were measured at age approximately 6-, 9- and 13-years during school hours by trained researchers. Height was measured to the nearest 1 mm by a Harpenden stadiometer. Body weight was measured to the nearest 0.1 kg by a SECA electronic scale (Seca 882, Medical Scales, NY, USA). Bicipital, tricipital, subscapular and suprailiac skinfolds were measured to the nearest 1 mm by a Harpenden skinfold calliper. The sum of four skinfolds (∑SF) was calculated by summarizing the four skinfold measurements [25]. Child BMI was calculated from weight in kilograms divided by height measured in meters squared [25]. Change in BMI and skinfold thickness (∆BMI/SF) between different ages was calculated by subtracting BMI or ∑SF at age 6 years from BMI or ∑SF at age 9 or 13 years.

The assessment of pubertal stages at age 9 and 13 years was identified using the picture scale by Tanner [22]. Pubertal development in girls was assessed based on breast development (b1 indicates undeveloped breasts and b5 indicates full/completed breast development) and pubic hair growth (ph1 indicates no pubic hair yet and ph5 indicates full pubic hair growth). The assessment in boys was based on genital development (g1 indicates that genital growth has not yet begun and g5 indicates that genital development is fully develop) and pubic hair growth (same as for girls). The children were asked to evaluate their stage of sexual maturity (with same-sex lab technicians) by comparison with Tanner's photos and drawings [22].

### Additional information

Physical activity was measured by the MTI 7164 activity monitor (Manufactory Technology Inc., Fort Walton Beach, FL, USA) [22] for four consecutive days (2 weekendays+2 weekdays) at age 6 years. The children were required to always wear the accelerometer, except during sleep and water-based activities. Zero counts (no activity) for 10 minutes or more were interpreted as "accelerometer not worn" and removed from the data [25]. Hence, 658 children that accumulated more than 8 hours of activity per day for at least 3 days were included in the analyses [25, 27].

Information on paternal and maternal weight and height was obtained by questionnaire at enrolment. Socioeconomic status (SES) categories were based on maternal education, as previous studies suggest closer relationship to children’s dietary habits compared to paternal education or income [28–30]. Information about maternal education level was obtained using two questions as previously described [26] and the level was divided into three categories:(1) short: completed elementary school (≤10 years) (2); medium: completed high school (12 years) or a short education (3 years) (3); long: completed college or university.

### Statistical analysis

Characteristics of the study participants were presented as medians and corresponding interquartile ranges (IQR) for continuous variables and as percentages for categorical variables. All statistical analyses were performed using SAS 9.4. Statistical significance was declared if a two-sampled p-value was < 0.05.

Linear regression models were used to investigate the association between intake of n-3 LCPUFA/TFA at age 6 years and changes in BMI or SF from age 6 to 9 years and age 6 to 13 years. We explored both the overall associations and substitution effects focusing on all other fats or specific subgroups of fat (SFAs, MUFAs and other PUFAs). To investigate substitution effects, we constructed regression models that included information on n-3 LCPUFA and all other energy given components of the diet, except for the nutrient to be substituted. The estimated corresponding regression coefficient would usually be interpreted as the effect of 1-unit n-3 LCPUFA replacing the omitted nutrient because all nutrients included in the model can be assumed constant. However, since we are using a cross-sectional measure of dietary intake, a more arcuate interpretation of the regression coefficient is the estimated difference between individuals in the dependent variable for a 1-unit higher intake of n-3 LCPUFA and a concomitantly lower intake of the omitted nutrient [31]. The substitution models were conducted defining the dietary intake as both absolute intake (g/day) and relative intake [energy percentages (E%)].

All results were presented using a three-step adjustment scheme. The crude model investigated the effect of n-3 LCPUFA/TFA intake or substituting other fats for n-3 LCPUFA on changes in BMI or SF, and adjusted for sex, age and the baseline BMI or SF. For the adjusted model, we further added information on baseline physical activity, intervention/comparison group, maternal and paternal BMI and maternal education. Finally, in a third model, we added information on total energy intake. Analyses both with and without adjustment for total energy intake were conducted to explore whether potential associations were mediated by or independent of total energy intake.

As sensitivity analysis we examined if children included in our analyses differed from the remaining children (those excluded due to missing information) participating in CoSCIS, differences in baseline characteristics were tested using Wilcoxon rank sum for continuous variables and Chi-squared test for categorical variables. Sensitivity analyses were made, including additional adjustment for pubertal stage, and for the associations between fatty acids intake and BMI also for changes over time in height.

## Result

In total, 285 children had complete data at follow-up after 3 years (aged 9 years) and 221 children had complete data at follow up after 7 years (aged 13 years) and were included in the analyses (Table 1). The majority of children had mothers with medium level education (completed high school); n=103, (36.1%) or long education (n=107, 37.5%), and 26.3% had mothers with short education.

**Table 1:** Characteristics of the participants at baseline and the follow-up examination

Characteristics	Median	Q1	Q3
Parental characteristics at baseline (n=285)			
Paternal BMI (kg/m2)	25.1	23.2	26.9
Maternal BMI (kg/m2)	22.8	20.8	25.2
Maternal education (n=285)			
Short n (%)	75 (26.3%)		
Medium n (%)	103 (36.1%)		
Long n (%)	107 (37.5%)		
Characteristics at baseline (6-years of age, n=285)			
Sex			
Boys (%)	130 (45.6%)		
Girls (%)	155 (54.4%)		
Weight (kg)	23.9	21.9	26.0
Height (cm)	123.0	119.8	125.5
Sum of skinfold (mm)^a^	24.4	20.8	28.9
BMI (kg/m^2^)	15.7	15.0	16.7
Physical activity score (cpm)	695.6	590.5	837.8
Energy intake (MJ/d)	8.2	7.2	9.2
Total fat (E%)	36.0	32.6	38.9
Total fat (g/d)	78.4	65.5	92.9
SFA (E%)	11.8	10.6	12.9
SFA (g/d)	26.1	21.2	31.0
MUFA (E%)	15.5	13.8	17.2
MUFA (g/d)	33.6	27.8	40.5
PUFA (E%)	5.0	4.4	5.5
PUFA (g/d)	10.9	9.3	13.4
n-3 LCPUFA (E%)	0.2	0.2	0.3
n-3 LCPUFA (g/d)	0.5	0.4	0.7
TFA (E%)	0.7	0.5	0.8
TFA (g/d)	1.5	1.1	1.9
Protein (E%)	14.0	12.8	15.2
Protein (g/d)	66.5	60.0	77.0
Carbohydrate (E%)	48.6	45.7	51.5
Carbohydrate (g/d)	253.2	222.3	285.8
Characteristics at follow-up at 9-year-of age (n=285)			
Weight (kg)	32.4	29.5	36.3
Height (cm)	139.0	135.4	142.8
Sum of skinfold (mm)	27.3	22.3	38.6
BMI (kg/m^2^)	16.9	15.7	18.2
Characteristics at second follow up at 13-years-of age (n=221)			
Weight (kg)	50.4	45.2	55.5
Height (cm)	163.4	158.5	168
Sum of skinfold (mm)^b^	29.4	23.4	40.9
BMI (kg/m^2^)	18.7	17.3	20.4

^a^n=283; ^b^ n=220

Their total fat intake at baseline was 78.4 g/d (IQR: 65.1 to 93.3), which corresponds to 36.0 E% (32.5 to 38.9%) and the intake of n-3 LCPUFA was 0.5 g/d (0.37 to 0.64), corresponding to 0.2 E% (0.2 to 0.3). The children’s BMI and SF at baseline were 15.7 kg/m^2^ (15.0 to 16.7) and 24.4 mm (20.8 to 28.9), respectively.

The included children differed from those who were not included with respect to gender and maternal education, e.g. there was a slightly higher percentage of girls (p=0.02) and longer maternal education among the included children (p=0.006). Apart from that there were no other overall differences at baseline between the children included and those not included in the analyses (Table S1).

Results from the regression analyses showed no significant associations in the crude analyses between intake of n-3 LCPUFA or TFA and ∆BMI over the following 3 and 7 years in models where intake was expressed as absolute intake or relative intake (Table 2). Further adjustment for baseline physical activity, intervention group, maternal and parental BMI and maternal education level gave essentially similar results, as did results after additional adjustment for energy intake (Table 2).

**Table 2:** The association (β and 95%CI) between TFA intake and n-3 LCPUFA intake at 6 years of age and subsequent 3- and 7-year change in BMI (kg/m^2^). Absolute intake (g/d) and relative intake models (E%).

	Grams per day (g/d)						Energy percentage (E%)					
	3-year changes in BMI (kg/m^2^) (n=285)			7-year changes in BMI (kg/m^2^) (n=221)			3-year changes in BMI (kg/m^2^) (n=285)			7-year changes in BMI (kg/m^2^) (n=221)		
	β	95% CI	P	β	95% CI	P	β	95% CI	P	β	95% CI	P
TFA												
Crude^1^	0.1	-0.8;0.3	0.25	0.1	-0.3;0.5	0.60	0.5	-0.1;1.1	0.12	0.9	-0.3;2.0	0.14
Adjusted (basic)^2^	0.1	-0.1;0.3	0.32	0.1	-0.3;0.5	0.62	0.4	-0.2;1.0	0.20	0.7	-0.4;1.9	0.21
Adjusted (+EI)^3^	0.2	-0.1;0.5	0.14	0.3	-0.2;0.8	0.26	0.4	-0.2;1.0	0.18	0.8	-0.3;2.0	0.16
n-3 LCPUFA												
Crude^1^	0.1	-0.4;0.6	0.76	-0.2	-1.1;0.7	0.69	0.01	-1.2;1.2	0.98	-0.1	-2.2;2.0	0.91
Adjusted (basic)^2^	0.01	-0.5;0.5	0.95	-0.4	-1.3;0.5	0.35	-0.2	-1.3,1.0	0.77	-0.8	-2.9;1.3	0.47
Adjusted (+EI)^3^	0.03	-0.5;0.6	0.92	-0.4	-1.3;0.6	0.42	-0.2	-1.4;1.0	0.76	-0.8	-2.9;1.3	0.45
Substitution of other fats with n-3 LCPUFA												
Crude^4^	0.1	-0.4;0.7	0.64	-0.1	-1.0;0.9	0.90	-0.2	-1.4;1.0	0.75	-0.4	-2.5;1.7	0.68
Adjusted (basic)^2^	0.02	-0.5;0.5	0.93	-0.4	-1.3;0.6	0.44	-0.4	-1.5;0.8	0.53	-1.0	-3.1;1.1	0.35
Adjusted (+EI)^3^	-0.03	-0.6;0.5	0.91	-0.4	-1.4;0.5	0.38	-0.4	-1.6;0.8	0.53	-1.0	-3.1;1.1	0.33
Substitution of SFA with n-3 LCPUFA												
Crude^5^	0.1	-0.4;0.6	0.76	-0.1	-1.0;0.9	0.87	-0.7	-2.1;0.7	0.31	-1.6	-3.9;0.8	0.20
Adjusted (basic)^2^	-0.001	-0.5;0.5	0.997	-0.4	-1.3;0.6	0.44	-0.9	-2.2;0.5	0.21	-1.8	-4.1;0.5	0.13
Adjusted (+EI)^3^	-0.2	-0.7;0.4	0.60	-0.6	-1.6;0.4	0.27	-0.8	-2.2;0.5	0.21	-1.8	-4.1;0.5	0.13
Substitution of MUFA with n-3 LCPUFA												
Crude^6^	-0.02	-0.6;0.5	0.94	-0.2	-1.2;0.7	0.62	-0.4	-1.6;0.8	0.52	-0.6	-2.7;1.5	0.57
Adjusted (basic)^2^	-0.1	-0.6;0.4	0.75	-0.5	-1.4;0.5	0.32	-0.5	-1.7;0.6	0.37	-1.1	-3.2;1.0	0.31
Adjusted (+EI)^3^	-0.1	-0.7;0.4	0.61	-0.5	-1.5;0.5	0.28	-0.5	-1.7;0.6	0.37	-1.1	-3.2;1.0	0.29
Substitution of other PUFAs with n-3 LCPUFA												
Crude^7^	0.1	-0.5;0.6	0.82	-0.1	-1.1;0.8	0.78	-0.3	-1.5;1.0	0.67	-0.5	-2.6;1.7	0.66
Adjusted (basic)^2^	-0.02	-0.5;0.5	0.95	-0.4	-1.3;0.5	0.40	-0.5	-1.7;0.7	0.40	-1.1	-3.2;1.1	0.32
Adjusted (+EI)^3^	-0.06	-0.6;0.5	0.83	-0.4	-1.4;0.5	0.38	-0.5	-1.7;0.7	0.40	-1.1	-3.2;1.0	0.31

^1^adjusted for baseline BMI, age and sex; ^2^ adjusted further for baseline physical activity, intervention group, maternal and parental BMI and maternal education level; ^3^ Model adjusted further for energy intake

By holding the total energy intake, it was assumed that the increase in n-3 LCPUFA resulted in a corresponding decrease in the intake of the fat component being replaced in each model. Substitution model adjusted for baseline BMI, age, sex, baseline intake of carbohydrate and protein, baseline intake of non-specified fat

^4^Substitution model excluded total fat intake from the model; ^5^ Substitution model excluded SFA intake from the model; ^6^ Substitution model excluded MUFA intake from the model; ^7^ Substitution model excluded PUFAs intake from the model.

Furthermore, we observed no significant associations for ∆BMI over 3 and 7 years, when all other fats, SFA, MUFA or PUFA were substituted for n-3 LCPUFA, neither before or after adjusting for covariates or with the additional adjustment for total energy intake (Table 2). We further adjusted for puberty stage and ∆height in these analyses as BMI during childhood is associated with height development [32]. We observed no significant associations for any of these models (Table S3).

Similarly, no associations were observed between intake of n-3 LCPUFA or TFA and ∆SF over 3 or 7 years in analysis with relative or absolute intake (Table 3). Finally, we further adjusted for pubertal stage in the analyses, as skinfold thickness increase substantially when children start puberty [33]. For both TFA and n-3 LCPUFA, the results from these analyses were essentially similar to the results from other models (Table S4).

**Table 3:** The association (β and 95%CI) between TFA intake and n-3 LCPUFA intake at 6 years of age and subsequent 3- and 7-year change in SF (mm) in relative intake models (E%).

	Grams per day (g/d)						Energy percentage (E%)					
	3-year changes in SF (mm) (n=283)			7-year changes in SF (mm) (n=219)			3-year changes in SF (mm) (n=283)			7-year changes in SF (mm) (n=219)		
	β	95% CI	P	β	95% CI	P	β	95% CI	P	β	95% CI	P
TFA												
Crude^1^	0.1	-1.3;1.6	0.85	-0.01	-2.9;2.9	0.99	1.2	-3.3;5.6	0.61	3.7	-5.0;12.4	0.40
Adjusted (basic)^2^	0.1	-1.4;1.5	0.90	0.1	-2.8;3.0	0.95	0.7	-3.7;5.0	0.77	3.5	-5.2;12.2	0.43
Adjusted (+EI)^3^	0.5	-1.4;2.5	0.59	1.2	-2.7;5.1	0.54	1.0	-3.6;5.5	0.68	4.3	-4.6;13.3	0.34
n-3 LCPUFA												
Crude^1^	2.0	-1.7;5.7	0.28	-0.4	-7.0;6.2	0.91	4.2	-4.6;13.0	0.35	1.6	-14.1;17.3	0.84
Adjusted (basic)^2^	1.6	-2.0;5.2	0.38	-2.2	-8.7;4.4	0.52	2.9	-5.7;11.6	0.50	-3.1	-18.8;12.3	0.70
Adjusted (+EI)^3^	2.2	-1.7;6.1	0.27	-1.7	-8.7;5.4	0.64	2.9	-5.8;11.6	0.51	-3.3	-19.0;12.4	0.68
Substitution of other fats with n-3 LCPUFA												
Crude^4^	2.3	-1.6;6.3	0.24	1.1	-6.0;8.2	0.77	3.0	-5.9;11.9	0.50	1.0	-14.9;16.8	0.90
Adjusted (basic)^2^	1.7	-2.2;5.6	0.39	-1.2	-8.1;6.0	0.75	1.6	-7.1;10.4	0.72	-3.2	-19.0;13.7	0.69
Adjusted (+EI)^3^	1.7	-2.2;5.6	0.40	-1.4	-8.6;5.7	0.69	1.6	-7.1;10.4	0.72	-3.4	-19.3;12.4	0.67
Substitution of SFA with n-3 LCPUFA												
Crude^5^	2.3	-1.6;6.3	0.24	1.0	-6.1;8.1	0.78	-2.3	-12.4;7.8	0.66	-11.2	-29.0;6.6	0.21
Adjusted (basic)^2^	1.7	-2.2;5.6	0.39	-1.2	-8.3;6.0	0.75	-3.1	-12.9;6.8	0.54	-12.5	-30.2;5.2	0.16
Adjusted (+EI)^3^	0.7	-3.5;5.0	0.74	-3.6	-11.3;4.1	0.36	-3.1	-13.0;6.8	0.54	-12.5	-30.2;5.2	0.17
Substitution of MUFA with n-3 LCPUFA												
Crude^6^	2.0	-2.0;6.0	0.32	0.2	-6.9;7.3	0.95	1.8	-7.1;10.8	0.69	-0.04	-15.9;15.9	0.99
Adjusted (basic)^2^	1.5	-2.4;5.4	0.45	-1.7	-8.8;5.5	0.64	0.8	-8.0;9.6	0.85	-3.6	-19.5;12.3	0.66
Adjusted (+EI)^3^	1.1	-2.8;5.1	0.58	-1.8	-9.0;5.4	0.62	0.8	-8.0;9.6	0.86	-3.8	-19.7;12.1	0.64
Substitution of other PUFAs with n-3 LCPUFA												
Crude^7^	2.3	-1.6;6.3	0.25	0.7	-6.4;7.8	0.85	1.8	-7.1;10.8	0.69	-0.04	-15.9;15.9	0.99
Adjusted (basic)^2^	1.7	-2.2;5.6	0.39	-1.4	-8.5;5.7	0.70	0.8	-8.0;9.6	0.85	-3.6	-19.5;12.3	0.66
Adjusted (+EI)^3^	1.6	-2.3;5.6	0.42	-1.3	-8.5;5.9	0.72	0.8	-8.0;9.6	0.86	-3.8	-19.7;12.1	0.64

^1^adjusted for baseline SF, age and sex; ^2^ adjusted further for baseline physical activity, intervention group, maternal and parental BMI and maternal education level; ^3^ Model adjusted further for energy intake

By holding the total energy intake, it was assumed that the increase in n-3 LCPUFA resulted in a corresponding decrease in the intake of the fat component being replaced in each model. Substitution crude model adjusted for baseline SF, age, sex, baseline intake of carbohydrate and protein, baseline intake of non-specified fat

^4^Substitution model excluded total fat intake from the model; ^5^ Substitution model excluded SFA intake from the model; ^6^ Substitution model excluded MUFA intake from the model; ^7^ Substitution model excluded PUFAs intake from the model.

## Discussion

We generally found no associations between intakes of n-3 LCPUFA or TFA at age 6 years and subsequent 3- or 7-years ∆BMI and SF. Moreover, statistical substitution of other fat or SFA, MUFA, or other PUFAs with n-3 LCPUFA, showed no associations with the development in BMI or SF.

To the best of our knowledge, this study is the first to examine prospective associations between n-3 LCPUFA with and without specified replacements and adiposity development among healthy children, so we cannot compare the observed results of lack of association with results from other similar studies on children. We identified one previous randomized weight development trial giving n-3 LCPUFA supplements to children, and this study found no effect [34]. Other trial results in children generally focused on weight loss among children with overweight, and found inconsistent results [35–37].

Our results in relation to TFA are in line with the results from a previously published prospective study among 668 low- and middle-income children aged 5 to 12 years that did also not observe an association between serum TFAs and subsequent weight gain [13]. Similarly, a previous case-control study found no differences in consumption of TFA between children with a normal weight or obesity [38]. Most previous studies were conducted among adults, and a systematic review concluded that among adults a high TFA intake may lead to a small extra weight gain, albeit based on few studies that were all conducted among US adults [39].

Our study has several strengths. First, the anthropometric measurements were taken by trained researchers rather than being assessed by parents and then self-reported. Second, repeated skinfold thickness measurements over 7 years are rare and are direct measures of subcutaneous fat. Third, our multivariate linear models were adjusted for a variety of confounders as well as included energy adjusted models. Finally, substitution analyses allowed us to investigate the impact of substituting energy from fats for n-3 LCPUFA.

The results are limited by the self-recorded diet registration with 7-day records, as this method, although generally considered among the best for diet assessment, may have some limitations for the collection of information about foods that are eaten with low frequency such as fish or seafood, which are the main sources of n-3 LCPUFA [40, 41]. However, the 7-day food records used in the CoSCIS study incorporated day-to-day variation across both weekdays and weekend days of one week and may therefore, be considered a suitable method to generally measure the dietary information of the children [25], although it may be a burden for the parents and require motivation and literary skills [42]. In the present study, slightly more children to better educated mothers participated, which may be a strength for data collection. However, as for other dietary instruments, the 7-day food records can involve reporting bias and misreporting [25, 43]. Foods with adverse health effect (e.g. SFA or TFA) are more likely to be under-reported, while those with beneficial health effects (e.g. n-3 LCPUFA) are more likely to be over-reported [44]. However, previous studies assessed the accuracy of the pre-printed record used in the present study, and the result showed only modest misreporting in children aged 7-8 years [26, 45]. In addition, different from many other countries, most Danish children bring packed lunch boxes from home to school, so the food purchased by children at school is limited, especially at a young age [26]. Thus, the parents would have had good knowledge of their children's intake during school hours. Furthermore, another limitation might be self-reported puberty stage, as children tend to overestimate their development in the early stages of maturation and underestimate it in the later stages [46]. However, in the analyses models we also adjusted for changes in height and baseline BMI or SF.

In the present study, the intake of n-3 LCPUFA defined less than 20 percent the BMI or SF variation at age 7 or 13 years. The lack of associations between intake of fats and adiposity in our study may also be due to low accuracy of the fatty acids information in relation to the quality of the fatty acid composition data in the nutritional tables [47]. Therefore, bias in the fatty acids intake estimates in the study may have attenuated potential associations between the specific fatty acid and the weight and adiposity development. Our analyses included several confounders, but some potential unmeasured confounding may still have remained, and some residual confounding is inevitable. Lack of control for those confounders may also have attenuated potential true associations [48].

The assessment of growth in weight and body fat is complicated by the age of the children in the present study. They were 6 years at baseline and thus, around the age of the adiposity rebound, where some may not have reached the turning point yet, while others have started to increase in BMI [49]. Also, after 3- and especially 7- years follow-up, the children were in the different stages of puberty [50], which may have resulted in large variation in height due to the pubertal growth spurt [51]. Uncertainty in the outcome assessment may thus have an effect on power and on the ability to detect potential associations. However, we adjusted our results by including change in height and puberty stage and found essentially similar results, suggesting that difference in puberty staging and change in height did not substantially confound our findings. The relatively small sample size may, and thus low power of the study, can have increased the risk of type II error and chance findings [52].

As shown in a previous study, the CoSCIS intervention did not have an overall effect on diet intake, except for a decrease in the intake of SFA among children of mothers with long education [26]. In the present study, the mothers of the children included in the analyses tended to be more educated than did mothers of those not included. This may reduce the generalizability of the results, but also highlights the importance of considering parental education in relation to children’s dietary intake and risk of obesity.

## Conclusion

This longitudinal study did not provide evidence to support that a high TFA or a low n-3 LCPUFA intake among 6 year-old children was related to subsequent 3 and 7 years increase in BMI or SF. Statistically substituting other fats for n-3 LCPUFA also did not influence 3- and 7-years weight development. Large prospective studies are needed to better understand the relationships between fatty acids intake and development of overweight and obesity during childhood.

## Supplementary Information

**Additional file 1.**

## Data Availability

The data presented in this study are available on request to bfh-dl-eek@regionh.dk. The data are not publicly available due to the participant’s privacy and data protection.
