# Supplementary material for: Intake of n-3 LCPUFA and trans-fatty acids is unrelated to development in body mass index and body fat among children
Source: BMC Nutr. 2022 Jan 3;8:1. doi: 10.1186/s40795-021-00493-5 (PMC8722340; doi:10.1186/s40795-021-00493-5)
Supplement: Supplementary file 1 — Additional file 1. [file 40795_2021_493_MOESM1_ESM.docx]

**Table S1.** The association (β and 95%CI) between TFA intake and n-3 LCPUFA intake at 6 years of age and subsequent 3- and 7-year change in BMI (kg/m^2^) in adjusted for delta height and puberty

|  | Grams per day (g/d) | | | | | | Energy percentage (E%) | | | | | |
| --- | --- | --- | --- | --- | --- | --- | --- | --- | --- | --- | --- | --- |
|  | 3-year changes (n=285) | | | 7-year changes (n=221) | | | 3-year changes (n=285) | | | 7-year changes (n=221) | | |
|  | β | 95% CI | P | β | 95% CI | P | β | 95% CI | P | β | 95% CI | P |
| TFA | | | | | | | | | | | | |
| Adjusted (basic+height+ puberty)^2^ | 0.1 | -0.1;0.3 | 0.20 | 0.1 | -0.3;0.5 | 0.55 | 0.4 | -0.2;1.1 | 0.16 | 0.6 | -0.5;1.8 | 0.26 |
| Adjusted (+EI+height+puberty)^3^ | 0.2 | -0.1;0.5 | 0.11 | 0.3 | -0.2;0.8 | 0.28 | 0.5 | -0.2;1.1 | 0.15 | 0.7 | -0.4;1.9 | 0.22 |
| n6/n3 ratio | | | | | | | | | | | | |
| Adjusted (basic+height+ puberty)^2^ | 0.1 | -0.3;0.4 | 0.72 | 0.5 | -0.1;1.1 | 0.11 | 0.1 | -0.3;0.4 | 0.72 | 0.5 | -0.1;1.1 | 0.11 |
| Adjusted (+EI+height+puberty)^3^ | 0.1 | -0.2;0.4 | 0.56 | 0.5 | -0.1;1.1 | 0.12 | 0.1 | -0.2;0.4 | 0.56 | 0.5 | -0.1;1.1 | 0.12 |
| n-3 LCPUFA | | | | | | | | | | | | |
| Adjusted  (basic+puberty+height)^1^ | 0.04 | -0.5;0.5 | 0.86 | -0.4 | -1.3;0.5 | 0.34 | -0.2 | -1.4,1.0 | 0.71 | -0.8 | -2.9;1.3 | 0.44 |
| Adjusted (+EI+height+puberty)^3^ | 0.04 | -0.5;0.6 | 0.87 | -0.4 | -1.4;0.5 | 0.35 | -0.2 | -1.4;1.0 | 0.72 | -0.8 | -2.9;1.2 | 0.43 |
| Substitution of n-3 LCPUFA with all other fats | | | | | | | | | | | | |
| Adjusted  (basic+puberty+height)^1^ | 0.04 | -0.5;0.6 | 0.89 | -0.4 | -1.3;0.5 | 0.40 | -0.4 | -1.6;0.8 | 0.50 | -1.0 | -3.0;1.1 | 0.35 |
| Adjusted (+EI+height+puberty)^3^ | -0.02 | -0.5;0.5 | 0.95 | -0.4 | -1.4;0.5 | 0.34 | -0.4 | -1.6;0.8 | 0.50 | -1.0 | -3.1;1.0 | 0.33 |
| Substitution of n-3 LCPUFA for SFA | | | | | | | | | | | | |
| Adjusted  (basic+puberty+height)^1^ | -0.01 | -0.5;0.5 | 0.96 | -0.4 | -1.3;0.5 | 0.40 | -0.8 | -2.1;0.6 | 0.25 | -1.7 | -4.0;0.6 | 0.15 |
| Adjusted (+EI+height+puberty)^3^ | -0.1 | -0.7;0.5 | 0.69 | -0.6 | -1.6;0.4 | 0.27 | -0.8 | -2.1;0.6 | 0.25 | -1.7 | -4.0;0.6 | 0.14 |
| Substituted of n-3 LCPUFA for MUFA | | | | | | | | | | | | |
| Adjusted  (basic+puberty+height)^1^ | -0.1 | -0.6;0.5 | 0.81 | -0.5 | -1.4;0.4 | 0.30 | -0.5 | -1.7;0.7 | 0.39 | -1.0 | -3.1;1.0 | 0.33 |
| Adjusted (+EI+height+puberty)^3^ | -0.1 | -0.6;0.4 | 0.71 | -0.5 | -1.5;0.4 | 0.29 | -0.5 | -1.7;0.7 | 0.39 | -1.1 | -3.1;1.0 | 0.31 |
| Substituted of n-3 LCPUFA for other PUFAs | | | | | | | | | | | | |
| Adjusted  (basic+puberty+height)^1^ | -0.01 | -0.5;0.5 | 0.98 | -0.4 | -1.4;0.5 | 0.35 | -0.5 | -1.7;0.7 | 0.43 | -1.0 | -3.1;1.1 | 0.34 |
| Adjusted (+EI+height+puberty)^3^ | -0.03 | -0.6;0.5 | 0.90 | -0.4 | -1.4;0.5 | 0.36 | -0.4 | -1.7;0.7 | 0.43 | -1.0 | -3.1;1.0 | 0.33 |

**Table S2.** The association (β and 95%CI) between TFA intake and n-3 LCPUFA intake at 6 years of age and subsequent 3- and 7-year change in SF (mm) in adjusted for puberty

|  | Grams per day (g/d) | | | | | | Energy percentage (E%) | | | | | |
| --- | --- | --- | --- | --- | --- | --- | --- | --- | --- | --- | --- | --- |
|  | 3-year changes in SF (mm) (n=285) | | | 7-year changes in SF (mm) (n=221) | | | 3-year changes in SF (mm) (n=285) | | | 7-year changes in SF (mm) (n=221) | | |
|  | Β | 95% CI | P | β | 95% CI | P | β | 95% CI | P | β | 95% CI | P |
| TFA | | | | | | | | | | | | |
| Adjusted (basic+ puberty)^2^ | 0.2 | -1.3;1.8 | 0.76 | 0.04 | -2.8;2.9 | 0.98 | 0.8 | -3.7;5.4 | 0.72 | 3.1 | -5.7;11.9 | 0.49 |
| Adjusted (+EI+ puberty)^3^ | 0.7 | -1.4;2.7 | 0.53 | 1.1 | -2.9;5.0 | 0.60 | 1.0 | -3.7;5.8 | 0.66 | 3.9 | -5.2;12.9 | 0.40 |
| n6/n3 ratio | | | | | | | | | | | | |
| Adjusted (basic+ puberty)^2^ | 0.6 | -1.9;3.0 | 0.65 | 2.6 | -1.9;7.2 | 0.25 | 0.6 | -1.9;3.0 | 0.65 | 2.6 | -1.9;7.2 | 0.25 |
| Adjusted (+EI+ puberty)^3^ | 0.7 | -1.7;3.2 | 0.55 | 2.5 | 2.0;7.1 | 0.28 | 0.7 | -1.7;3.2 | 0.55 | 2.5 | 2.0;7.1 | 0.28 |
| n-3 LCPUFA | | | | | | | | | | | | |
| Adjusted (basic+ puberty)^2^ | 1.4 | -2.3;5.0 | 0.46 | -2.3 | -8.8;4.3 | 0.50 | 1.7 | -7.1;10.5 | 0.70 | -3.3 | -19.0;12.3 | 0.67 |
| Adjusted (+EI+ puberty)^3^ | 1.7 | -2.2;5.7 | 0.38 | -1.9 | -8.9;5.2 | 0.60 | 1.7 | -7.1;10.5 | 0.70 | -3.5 | 19.3;12.2 | 0.66 |
| Substitution of n-3 LCPUFA with all fats | | | | | | | | | | | | |
| Adjusted (basic+ puberty)^2^ | 1.3 | -2.6;5.2 | 0.52 | -1.3 | -8.4;5.8 | 0.72 | 0.5 | -8.3;9.3 | 0.92 | -3.3 | -19.1;12.6 | 0.68 |
| Adjusted (+EI+ puberty)^3^ | 1.3 | -2.7;5.2 | 0.53 | -1.5 | -8.7;5.6 | 0.67 | 0.5 | -8.4;9.3 | 0.92 | -3.5 | -19.4;12.3 | 0.66 |
| Substitution of n-3 LCPUFA for SFA | | | | | | | | | | | | |
| Adjusted (basic+ puberty)^2^ | 1.3 | -2.6;5.2 | 0.52 | -1.3 | -8.4;5.9 | 0.72 | -3.7 | -13.6;6.3 | 0.47 | -12.3 | -30.0;5.5 | 0.17 |
| Adjusted (+EI+ puberty)^3^ | 0.4 | -3.9;4.7 | 0.86 | -3.5 | -11.2;4.1 | 0.36 | -3.7 | -13.6;6.3 | 0.47 | -12.2 | -30.0;5.5 | 0.18 |
| Substitution of n-3 LCPUFA for MUFA | | | | | | | | | | | | |
| Adjusted (basic+ puberty)^2^ | 1.1 | -2.8;5.1 | 0.58 | -1.8 | -9.0;5.4 | 0.62 | -0.1 | -9.0;8.8 | 0.98 | -3.7 | -19.6;12.2 | 0.65 |
| Adjusted (+EI+ puberty)^3^ | 0.9 | -3.2;4.9 | 0.67 | -1.9 | -9.1;5.3 | 0.60 | -0.1 | -9.0;8.8 | 0.98 | -3.9 | -19.8;12.0 | 0.63 |
| Substitution of n-3 LCPUFA for other PUFAs | | | | | | | | | | | | |
| Adjusted (basic+ puberty)^2^ | 1.3 | -2.7;5.2 | 0.53 | -1.5 | -8.7;5.6 | 0.68 | -0.2 | -9.2;8.8 | 0.97 | -3.3 | -19.4;12.8 | 0.69 |
| Adjusted (+EI+ puberty)^3^ | 1.3 | -2.7;5.2 | 0.53 | -1.4 | -8.6;5.7 | 0.70 | -0.2 | -9.2;8.9 | 0.97 | -3.4 | -19.5;12.7 | 0.67 |

**Table S3.** The association between n-3 LCPUFA intake at 6 years of age and subsequent 3- and 7-year change in BMI (kg/m^2^)

|  | Grams per day (g/d) | | | | | | | | Energy percentage (E%) | | | | | | | |
| --- | --- | --- | --- | --- | --- | --- | --- | --- | --- | --- | --- | --- | --- | --- | --- | --- |
|  | 3-year changes in BMI (kg/m^2^) (n=285) | | | | 7-year changes in BMI (kg/m^2^) (n=221) | | | | 3-year changes in BMI (kg/m^2^) (n=285) | | | | 7-year changes in BMI (kg/m^2^) (n=221) | | | |
|  | β | 95% CI | P | R^2^ | β | 95% CI | P | R^2^ | β | 95% CI | P | R^2^ | β | 95% CI | P | R^2^ |
| n-3 LCPUFA | | | | | | | | | | | | | | | | |
| Crude^1^ | 0.1 | -0.4;0.6 | 0.76 | 0.12 | -0.2 | -1.1;0.7 | 0.69 | 0.13 | 0.01 | -1.2;1.2 | 0.98 | 0.12 | -0.1 | -2.2;2.0 | 0.91 | 0.13 |
| Adjusted (basic)^2^ | 0.01 | -0.5;0.5 | 0.95 | 0.17 | -0.4 | -1.3;0.5 | 0.35 | 0.19 | -0.2 | -1.3,1.0 | 0.77 | 0.17 | -0.8 | -2.9;1.3 | 0.47 | 0.19 |
| Adjusted (+EI)^3^ | 0.03 | -0.5;0.6 | 0.92 | 0.17 | -0.4 | -1.3;0.6 | 0.42 | 0.19 | -0.2 | -1.4;1.0 | 0.76 | 0.17 | -0.8 | -2.9;1.3 | 0.45 | 0.20 |

**Table S4.** The association between n-3 LCPUFA intake at 6 years of age and subsequent 3- and 7-year change in SF (mm)

|  | Grams per day (g/d) | | | | | | | | Energy percentage (E%) | | | | | | | |
| --- | --- | --- | --- | --- | --- | --- | --- | --- | --- | --- | --- | --- | --- | --- | --- | --- |
|  | 3-year changes in BMI (kg/m^2^) (n=285) | | | | 7-year changes in BMI (kg/m^2^) (n=221) | | | | 3-year changes in BMI (kg/m^2^) (n=285) | | | | 7-year changes in BMI (kg/m^2^) (n=221) | | | |
|  | β | 95% CI | P | R^2^ | β | 95% CI | P | R^2^ | β | 95% CI | P | R^2^ | β | 95% CI | P | R^2^ |
| n-3 LCPUFA | | | | | | | | | | | | | | | | |
| Crude^1^ | 2.0 | -1.7;5.7 | 0.28 | 0.20 | -0.4 | -7.0;6.2 | 0.91 | 0.10 | 4.2 | -4.6;13.0 | 0.35 | 0.20 | 4.2 | -4.6;13.0 | 0.35 | 0.10 |
| Adjusted (basic)^2^ | 1.6 | -2.0;5.2 | 0.38 | 0.25 | -2.2 | -8.7;4.4 | 0.52 | 0.15 | 2.9 | -5.7;11.6 | 0.50 | 0.25 | 2.9 | -5.7;11.6 | 0.50 | 0.15 |
| Adjusted (+EI)^3^ | 2.2 | -1.7;6.1 | 0.27 | 0.25 | -1.7 | -8.7;5.4 | 0.64 | 0.15 | 2.9 | -5.8;11.6 | 0.51 | 0.25 | 2.9 | -5.8;11.6 | 0.51 | 0.15 |
